# LUD, a new protein domain associated with lactate utilization

**DOI:** 10.1186/1471-2105-14-341

**Published:** 2013-11-26

**Authors:** William C Hwang, Constantina Bakolitsa, Marco Punta, Penelope C Coggill, Alex Bateman, Herbert L Axelrod, Neil D Rawlings, Mayya Sedova, Scott N Peterson, Ruth Y Eberhardt, L Aravind, Jaime Pascual, Adam Godzik

**Affiliations:** 1 Joint Center for Structural Genomics, La Jolla, CA 92037, USA; 2 Sanford Burnham Medical Research Institute, 10901 North Torrey Pines Road, La Jolla, CA 92037, USA; 3 Wellcome Trust Sanger Institute, Wellcome Trust Genome Campus, Hinxton, Cambridgeshire CB10 1SA, UK; 4 European Molecular Biology Laboratory, European Bioinformatics Institute, Wellcome Trust Genome Campus, Hinxton, Cambridgeshire CB10 1SD, UK; 5 Stanford Synchrotron Radiation Lightsource, SLAC National Accelerator Laboratory, Menlo Park, CA 94025, USA; 6 National Center for Biotechnology Information, National Institutes of Health, Bethesda, MD 20894, USA; 7 Department of Molecular and Experimental Medicine, The Scripps Research Institute, La Jolla, CA 92037, USA; 8 Center for Research in Biological Systems, University of California, San Diego, La Jolla, CA 92093‑0446, USA; 9 Center of Excellence in Genomic Medicine Research, King Abdulaziz University, Jeddah 21589, Kingdom of Saudi Arabia

**Keywords:** LUD, DUF162, LutB, LutC, Domain of unknown function, *Deinococcus radiodurans*

## Abstract

**Background:**

A novel highly conserved protein domain, DUF162 [Pfam: PF02589], can be mapped to two proteins: LutB and LutC. Both proteins are encoded by a highly conserved LutABC operon, which has been implicated in lactate utilization in bacteria. Based on our analysis of its sequence, structure, and recent experimental evidence reported by other groups, we hereby redefine DUF162 as the LUD domain family.

**Results:**

JCSG solved the first crystal structure [PDB:2G40] from the LUD domain family: LutC protein, encoded by ORF *DR_1909*, of *Deinococcus radiodurans*. LutC shares features with domains in the functionally diverse ISOCOT superfamily. We have observed that the LUD domain has an increased abundance in the human gut microbiome.

**Conclusions:**

We propose a model for the substrate and cofactor binding and regulation in LUD domain. The significance of LUD-containing proteins in the human gut microbiome, and the implication of lactate metabolism in the radiation-resistance of *Deinococcus radiodurans* are discussed.

## Background

We are now in an era when we can routinely sequence the complete genomes of microbes and rapidly identify their protein coding complements. The sequences of millions of proteins are now known. Despite this wealth of information we are still far from understanding how all of these proteins operate to give rise to a living organism. At present, in a consistent percentage of proteins the predicted function remains unknown [1,2]. From our analysis of 23 million proteins in the Pfam sequence database (Pfam release 27.0), 20% of them have no associated Pfam domain [3] and more are classified into DUF (Domains of Unknown Function) families [2]. This uncharacterized set of proteins potentially contains novel biological systems. Therefore, it is important to uncover these hidden functions through analysis of protein sequence, protein structure, and finally through directed experimental analyses [4-7].

There have been various attempts to classify the multitude of protein sequences into families to facilitate an improved understanding of the functional repertoire of proteins. In addition, there is a growing number of protein families defined for which no protein has ever been previously experimentally characterized. These families have been called DUFs [2] or Uncharacterized Protein Families (UPFs) [8]. The Pfam database contains one of the largest collections of such families with over 4,000 defined to date.

A novel domain, DUF162 [Pfam: PF02589] [COG: COG1556] [eggNOG: COG1556] [CDD: 224473], was found predominantly in Bacteria, and to a lesser extent in Archaea and Eukaryota. Recently, one protein (YvbY from *Bacillus subtilis*) in this DUF162 family was identified as lactate-utilization protein C (LutC), which was homologous to the YkgG protein in *E. coli*, hinting at a possible role in lactate utilization [9,10]. Indeed, DUF162 domain is a constituent domain of two proteins (LutB and LutC) encoded by the conserved LutABC operon in bacteria. This operon has been linked to lactate utilization [9,10] and is implicated in the oxidative conversion of L-lactate into pyruvate [9]. Based on our analysis of its sequence, structure, and recent experimental evidence reported by other groups, we hereby redefine DUF162 domain as the LUD domain.

Here, we report the first crystal structure [PDB: 2G40] of the LUD domain family: LutC protein (encoded by ORF DR_1909) from *Deinococcus radiodurans*[11,12] at 1.70 Å resolution. We propose a model for the substrate and cofactor binding and regulation.

## Results and discussion

### LUD domain structure

**Figure 1:**
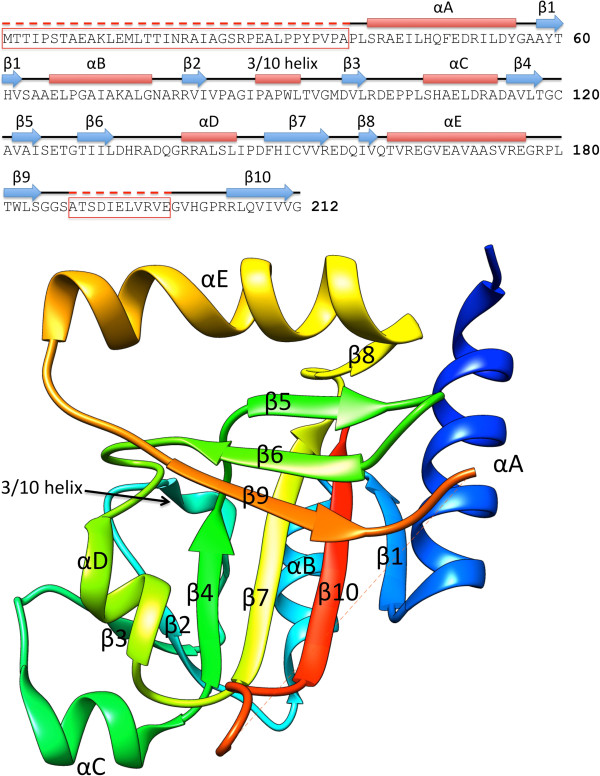
**Structure of LutC protein from *Deinococcus radiodurans*.** The protein structure is shown in cartoon style and colored in rainbow format (N-terminus being blue and C-terminus red). The dashed line in the figure represents a break in the protein polypeptide chain as a result of missing electron density in the protein structure.

The Joint Center for Structural Genomics (JCSG) determined the first crystal structure of the LUD domain family: LutC protein from *Deinococcus radiodurans*. The LutC protein structure is a mixed alpha-helix and beta-sheet protein (Figure 1). The protein core is made up of two orthogonal beta-sheets, each consisting of four beta-strands. The alpha-helices are packed against the two solvent-facing surfaces of the beta-sheets as well as against the side openings of the protein core.

**Figure 2:**
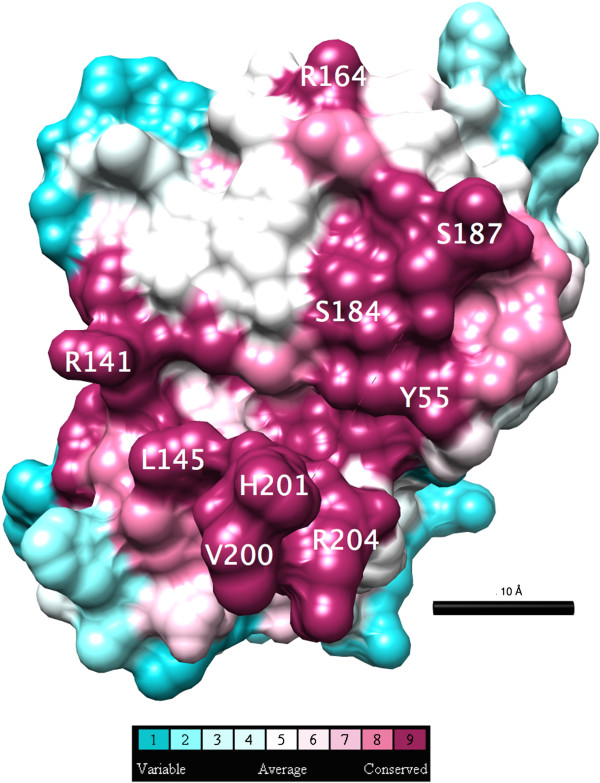
**Conservation of residues in the LUD domain family projected on the three dimensional structure of the LutC protein from *Deinococcus radiodurans*.**

Some regions of the LutC protein sequence are highly conserved as assessed by ConSurf. The conserved areas are concentrated on one side of the structure and form a groove about 20 Å in length (Figure 2), which might be functionally important. LutC protein appears to be dimeric, with a buried surface of 1721 Å^2^ at the dimer-interface. The highly conserved area coincides with parts of the dimer interface.

Structural alignment with other protein structures present in the Protein Data Bank, using the program DALI [13,14], suggests LutC protein is structurally akin to proteins found in the ISOCOT superfamily [15]. This is consistent with its classification in SCOP [16] as part of the NagB/RpiA/CoA transferase-like fold and superfamily. The ISOCOT superfamily is known to comprise proteins of diverse functions including sugar isomerases, translation factor eIF2B, ligand-binding domains of the DeoR-family transcription factors, acetyl-CoA transferases, and methenyltetrahydrofolate synthetase [15].

### Domain organization

**Figure 3:**
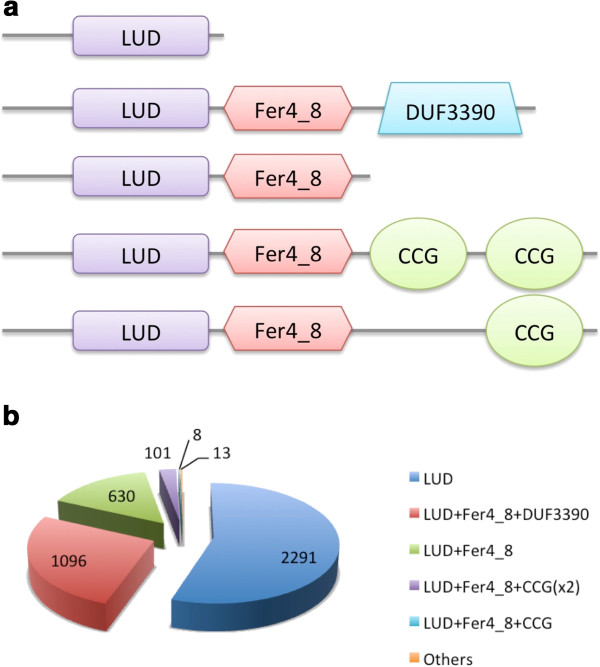
**Domain organization of LUD domain. a**. The most common domain organizations of LUD domain are shown. While predominantly found to exist by itself, LUD domain is also frequently found together with domains such as 4Fe-4S dicluster domain Fer4_8 [Pfam:PF13183], DUF3390 [Pfam:PF11870], cysteine-rich iron-sulfur binding cluster domain CCG [Pfam:PF02754]. **b**. Pie chart showing the frequency of common LUD domain organizations in known proteins.

While predominantly found to exist by itself, LUD domain is also frequently found together with domains such as the 4Fe-4S dicluster domain Fer4_8 [Pfam: PF13183], DUF3390 [Pfam: PF11870], and cysteine-rich iron-sulfur binding cluster domain CCG [Pfam: PF02754] [17]. Figure 3 shows the most common domain architectures featuring the LUD domain according to Pfam release 27.0.

### LUD domain-containing proteins encoded by the highly conserved LutABC Operon

**Figure 4:**
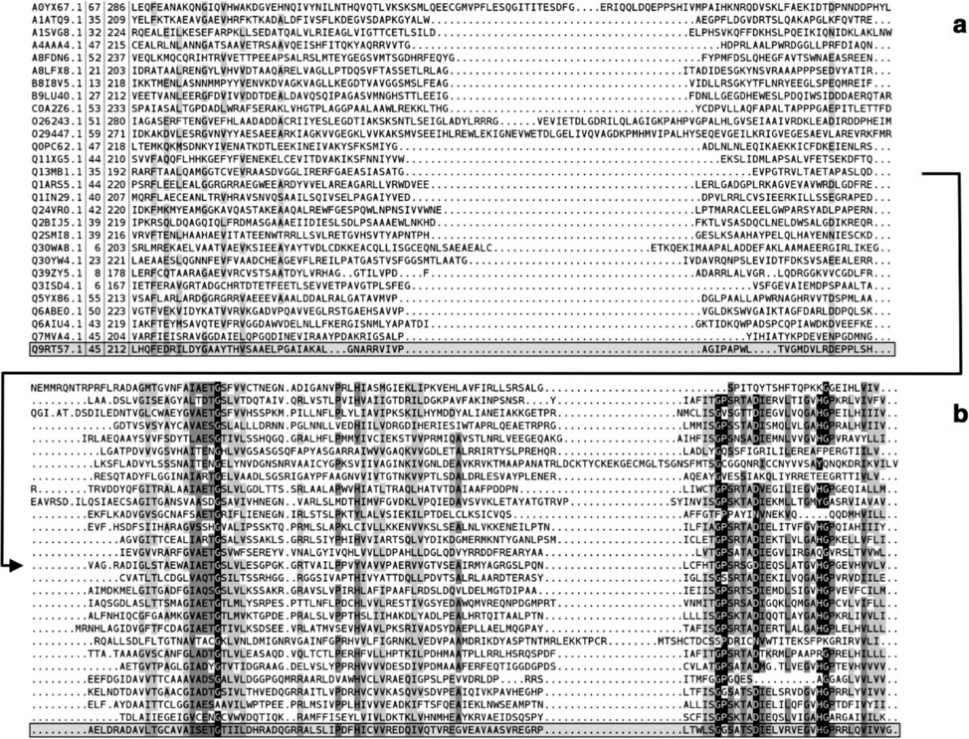
**Alignment with representative sequences of LUD family (Pfam DUF162-PF02589). a**. N-terminal part of the alignment. **b**. C-terminal part of the alignment. Shades of grey reflect average similarity.

**Figure 5:**
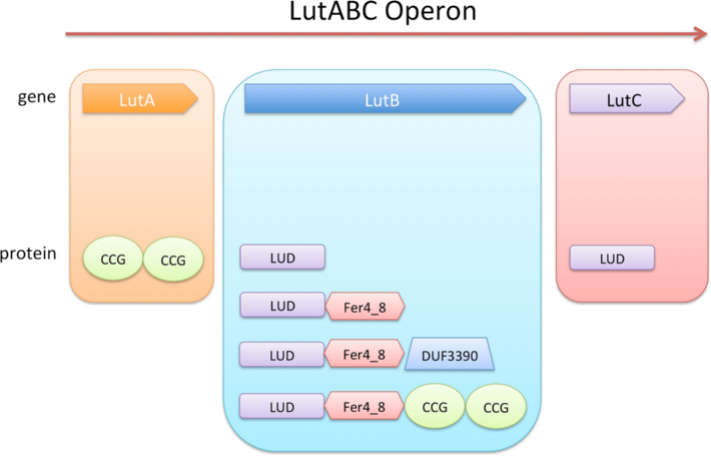
**Gene and protein make-up of the three elements of the LutABC operon.** The three genes making up the LutABC operon and the corresponding various proteins with their Pfam domains marked are shown.

**Figure 6:**
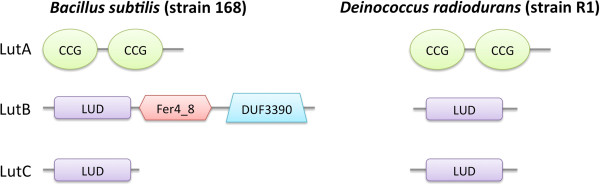
**LutABC domain organizations in *Bacillus subtilis *** **(strain 168) and *Deinococcus radiodurans *** **(strain R1).**

LUD domain is a protein domain of approximately 160 residues in length (Figure 4, and Additional file 1). It is found in two proteins encoded by the highly conserved LutABC operon (Figures 5 and 6), which appears in a wide variety of Gram-positive and Gram-negative bacteria [9]. The LutABC operon was found to be important for growth and biofilm formation in *Bacillus subtilis*[9]. The LUD domain is found in both LutB and LutC proteins encoded by the LutABC operon. In the vast majority of cases, the LUD domain is the only constituent domain of LutC proteins, whereas in LutB proteins it is often associated with protein families Fer4_8, CCG, or DUF3390 (Figure 5). Indeed, in Pfam release 27.0 there is just one instance of LutB protein being made of DUF162 alone, which occurs in *Deinococcus radiodurans* (Figure 6). However, searching the section of DNA in *Deinococcus radiodurans* from the start of *lutB* to the start of *lutC* finds a frame-shift and a copy of DUF3390 on the opposite strand, though no apparent Fer4_8, implying possible poor quality sequencing in this region. Finally, LutA protein is most often made of two copies of CCG domains. Both Fer4_8 and CCG domains are likely iron-sulfur cluster binding domains [17]. LutA protein is a putative iron-sulfur heterodisulfide reductase; LutB protein a putative iron-sulfur oxidoreductase; LutC protein a putative subunit of an iron-sulfur protein. Together, they are thought to mediate the oxidation of lactate via a cytochrome-like electron transfer chain, though the precise roles played by LutABC remain unclear [9].

### Presence in gut microbiome

It is worth noting that LUD domain has an increased abundance in gut microbiome. From our comparative genomics analysis of the metahit human gut microbiome of 124 human subjects (unpublished result, data not shown), the average ratio of number of homologs from the metahit human gut microbiome versus those found in UniProtKB is about 0.07. The ratio for LUD domain is ten times higher at 0.72, suggesting it plays a significant role in the gut microbiome, possibly related to its role in anaerobic metabolism. Interestingly, lactic acid bacteria (LAB) are being used as probiotics [18]. Lactate metabolism is integral to human health and host-pathogen interactions. Pathogenic bacteria have been shown to decrease local pH in hosts, through an increase in lactate production, so as to facilitate the release of iron from host transferrin [19]. In other species, acquisition of lactate is necessary for bacteremia [20] and colonization [21]. Lactate is also a potent signaling molecule in inflammatory pathways and has emerged as a critical regulator of cancer development, maintenance and metastasis [22]. By modulating lactate concentrations in the host’s environment through LUD domains and other lactate-related pathways, lactobacilli could thus influence the outcomes of both pathogenicity and disease [23].

### Model for LUD domain substrate-cofactor binding and regulation

**Figure 7:**
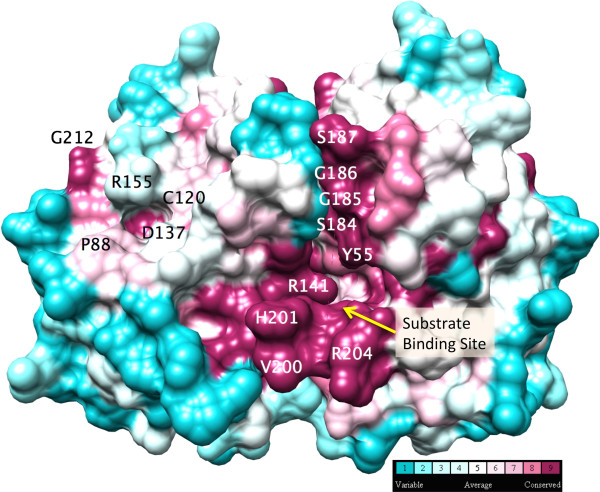
The highly conserved cavity near the dimer interface as the possible active site.

**Figure 8:**
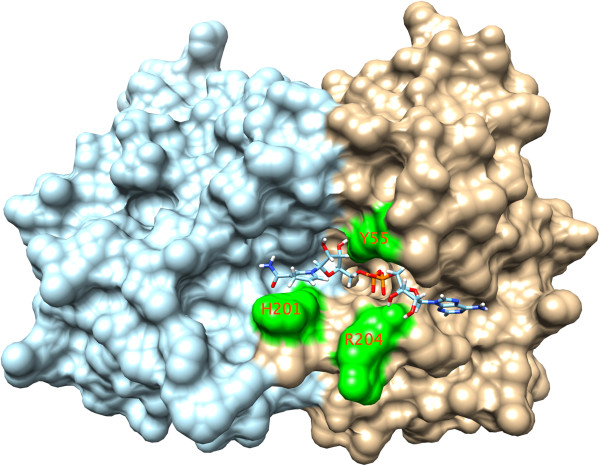
**Docking of NADH to the hypothetical active site near the dimer interface.** The monomers are colored in cyan and brown, respectively. Highly conserved residues, Y55, H201, R204 nearby are highlighted in green and labeled.

Inspection of the LutC protein dimer structure identified a highly conserved cavity (lined by residues Y55, H201, and R204) near the dimer interface. We proposed this cavity to be the putative active site (Figure 7), where the oxidative conversion of lactate into pyruvate occurs [9], based on the following observations: First, the residues surrounding this cavity are highly conserved, suggesting they are functionally important. Second, this cavity is large enough to accommodate both NAD + and lactate, hypothetical cofactor and substrate (Figure 8). NAD is among the top 5 possible ligands for LutC dimer as predicted by IsoCleft [24]. Top ligand predicted by Isocleft predicted was NDP (NADPH). Third, in the docking model the highly conserved H201 in LutC protein is located close to the substrate-cofactor reaction site and could hence serve as the catalytic histidine. Fourth, the 11-residue disordered loop (between S187 and G199) near this cavity could function as a substrate binding regulator, analogous to the role played by the disordered loop in the active site of lactate dehydrogenase (LDH), which converts pyruvate to lactate [25]. Taken together, it is likely that this pocket is indeed the active site.

Another moderately conserved cavity lined by residues R155, C120, and D137 (Figure 7), roughly coincides with the ISOCOT superfamily primary binding site. Docking of NAD to this shallow and small cavity leaves it not fully embedded and partially exposed. Thus, it is unlikely to form the active site. Nevertheless, this cavity could bind smaller molecules and is a good candidate for allosteric regulation. Allosteric regulation has been reported for certain proteins of the ISOCOT superfamily [26,27].

### Functional implications in Deinococcus radiodurans

The LutC protein was selected as a target because of the interest in *Deinococcus radiodurans by* JCSG. *Deinococcus radiodurans* is the most radiation-resistant bacterium known to date [12]. It can survive 4000 Gray (Gy) of irradiation, a dose hundreds of times greater than that considered lethal for most organisms. How it accomplishes such a remarkable feat remains enigmatic. A study examining global gene expression following ionizing radiation exposure and desiccation allowed a dissection of the response to double strand breaks (induced by both ionizing radiation and desiccation) and oxidative stress associated with reactive oxygen species (ROS). LutC protein was not induced in either treatment but was constitutively expressed [11]. Free radicals, in particular ROS, generated when cells are exposed to ionizing radiation, are cytotoxic. The unpaired electrons of free radicals render them highly reactive with biological molecules. Unsaturated fatty acids present in the membrane are particularly susceptible to free radicals. Furthermore, free radical-oxygen will deplete oxygen in the cytosol and abolish aerobic metabolism. Anaerobic lactate metabolism can be an indispensable alternative energy source. Moreover, lactate can function as a scavenger of free radicals [28]. Thus, lactate utilization may contribute to the radiation-resistance of the *Deinococcus radiodurans*. As the LutC protein from *Deinococcus radiodurans* represents a prototypical LUD domain in lactate utilization, it could be contributing towards radiation-resistance in this bacterium.

## Conclusions

Lactate metabolism is integral to human health, and may play a role in the radiation resistance in *Deinococcus radiodurans*. The LUD domain is a highly conserved protein domain that has recently been identified to play a role in lactate metabolism. In this report, we described the crystal structure of the *Deinococcus radiodurans* LutC protein, the first for a member of the LUD domain family. Using sequence and structure analysis, we proposed a model for the substrate and cofactor binding and regulation in LUD domains. We also analyzed possible implications for radiation resistance in *Deinococcus radiodurans*. Further experimental characterization will be needed to test these hypotheses.

## Methods

### Sequence analysis

Alignment of representative sequences of LUD family (Pfam DUF162-PF02589) was built by taking the SEED sequences of the family, reducing redundancy at 40% sequence identity and finally realigning the remaining sequences plus the sequence of 2G40 (UniProtKB id: Q9RT57) with ClustalW [29]. For better visualisation the alignment has been split in two parts (a) and (b). In (a) we show the N-terminal part of the alignment that continues toward the C-terminus in (b). Shades of grey reflect average similarity as calculated from the BLOSUM62 amino acid substitution matrix (black most conserved, white least conserved). Dashes (-) represent deletions, dots (.) represent insertions and lower case letters represent inserted residues. For each sequence, we report the UniProtKB id (e.g. F9YU00), the position along the protein sequence of first and last residue in the alignment (in the case of Q9RT57, for example, aligned residues range from 45 to 212) and, finally, the amino acid sequence. 2G40 (Q9RT57) sequence is highlighted by a shaded box. The alignment is visualized with Belvu [30] (sonnhammer.sbc.su.se/Belvu.html). More sequence and domain analysis for the LUD domain family can be found in the Additional file 1.

### Structure determination

Structure determination of LutC protein was carried out by the JCSG high-throughput structural biology pipeline [31]. Diffraction data were collected at Stanford Synchrotron Radiation Lightsource (SSRL) beamline 1-5. The crystal structure was determined by MAD phasing using seleno-methionine-derivatized protein. The structure was validated using the JCSG Quality Control server (http://smb.slac.stanford.edu/jcsg/QC). Experimental details as well as structural and refinement statistics can be found in the Additional file 2.

Atomic coordinates and experimental structure factors have been deposited into the Protein Data Bank (http://www.rcsb.org) with PDB ID: 2G40.

### Structure analysis

LutC protein dimer was generated by symmetry-related positions in Pymol [32]. Dimer interface was assessed by PISA [33]. Conservation of LutC protein amino acid residues was assessed by ConSurf [34], which obtained close homologous sequences through BLAST. Molecular docking was performed with MVD [35] using default parameters. Structure graphics were prepared in Chimera [36].

## Competing interests

The authors declare that they have no competing interests.

## Authors’ contributions

WH conceived the article and prepared the manuscript. AB wrote part of the introduction; MP and PC performed LUD domain family sequence alignment and domain analysis; MS performed the analysis of proteins with known versus unknown functions; SP contributed the discussion section on lutC gene expression following exposure to ionizing radiation; AB, MP, NR, PC, MS, SP, RE, AL, JP, CB, AG commented on the manuscript; HA prepared the experimental details of structure determination and refinement statistics for 2G40 in the supplementary information; JP and CB annotated the 2G40 structure on TOPSAN. All authors read and approved the final manuscript.

## Supplementary Material

Additional file 1 **Sequence and Domain Analysis.** This section contains additional sequence and domain analysis of LUD domain family. Click here for file

Additional file 2 **Experimental Details [PDB:2G40].** This section contains experimental details as well as structural and refinement statistics. Click here for file
