# Supplementary material for: LUD, a new protein domain associated with lactate utilization
Source: BMC Bioinformatics. 2013 Nov 26;14:341. doi: 10.1186/1471-2105-14-341 (PMC3924224; doi:10.1186/1471-2105-14-341)
Supplement: Additional file 1 — Sequence and Domain Analysis. This section contains additional sequence and domain analysis of LUD domain family. [file 1471-2105-14-341-S1.docx]

Additional file 1

**Figure 1.** The amino acid sequence of PDB 2G40 was used in the search for LUD domain using NCBI BLAST[[1](#_ENREF_1)]. The top 10 sequences annotated as LutC protein were retrieved and aligned with 2G40 using MAFFT[[2](#_ENREF_2)]. Aligned sequences were drawn in Jalview[[3](#_ENREF_3)]. The Refseq accession number for LutA, LutB, LutC in *Bacillus subtilis* (strain 168) are NP_391285, NP_391284, NP_391283, respectively; the Refseq accession number for LutA, LutB, LutC in *Deinococcus radiodurans* are NP_295630, NP_295631, NP_295632, respectively. The conserved residues labeled in Figure 2 in the main text are now highlighted in this figure with red dots above the sequence.


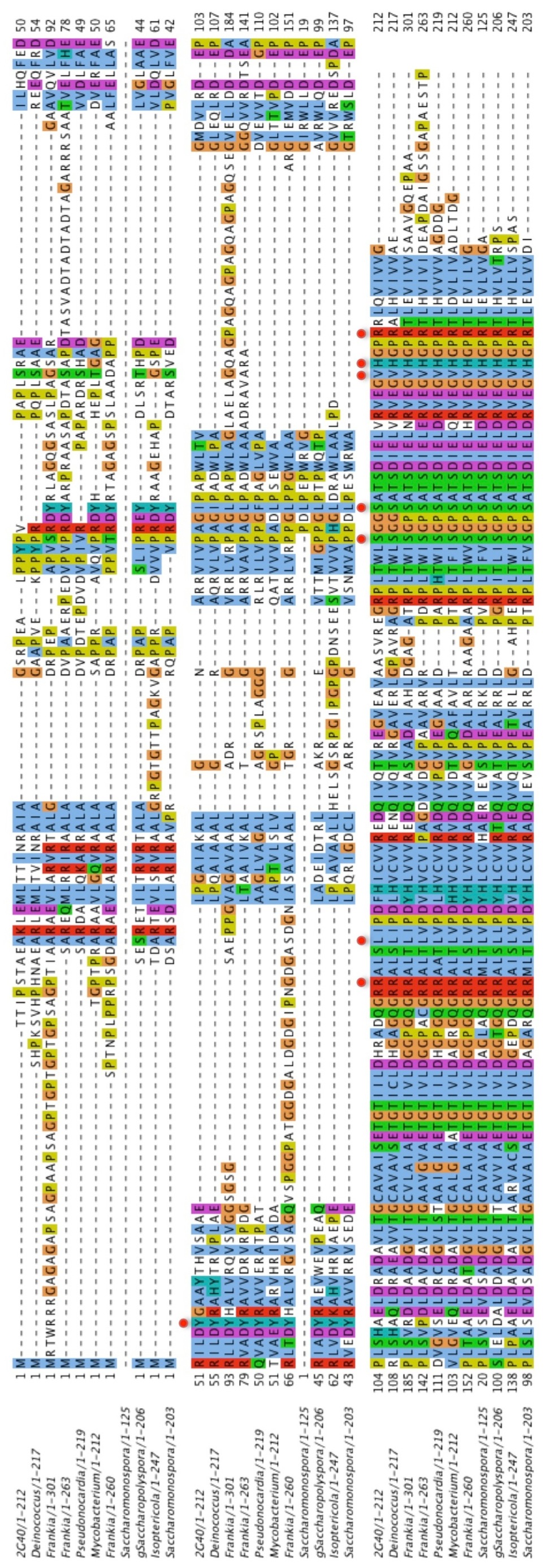


**Figure 2.** Genomic environments of LutABC operon. Genomic environments for the LutABC operon and surrounds were generated from the Microbesonline website[[4](#_ENREF_4)] in tree-form to show the seven closest matches. The starting gene for a) was from *Deinococcus radiodurans* DR_1909, and for b) was from *Bacillus subtilis* str 168 BSU34030.
**
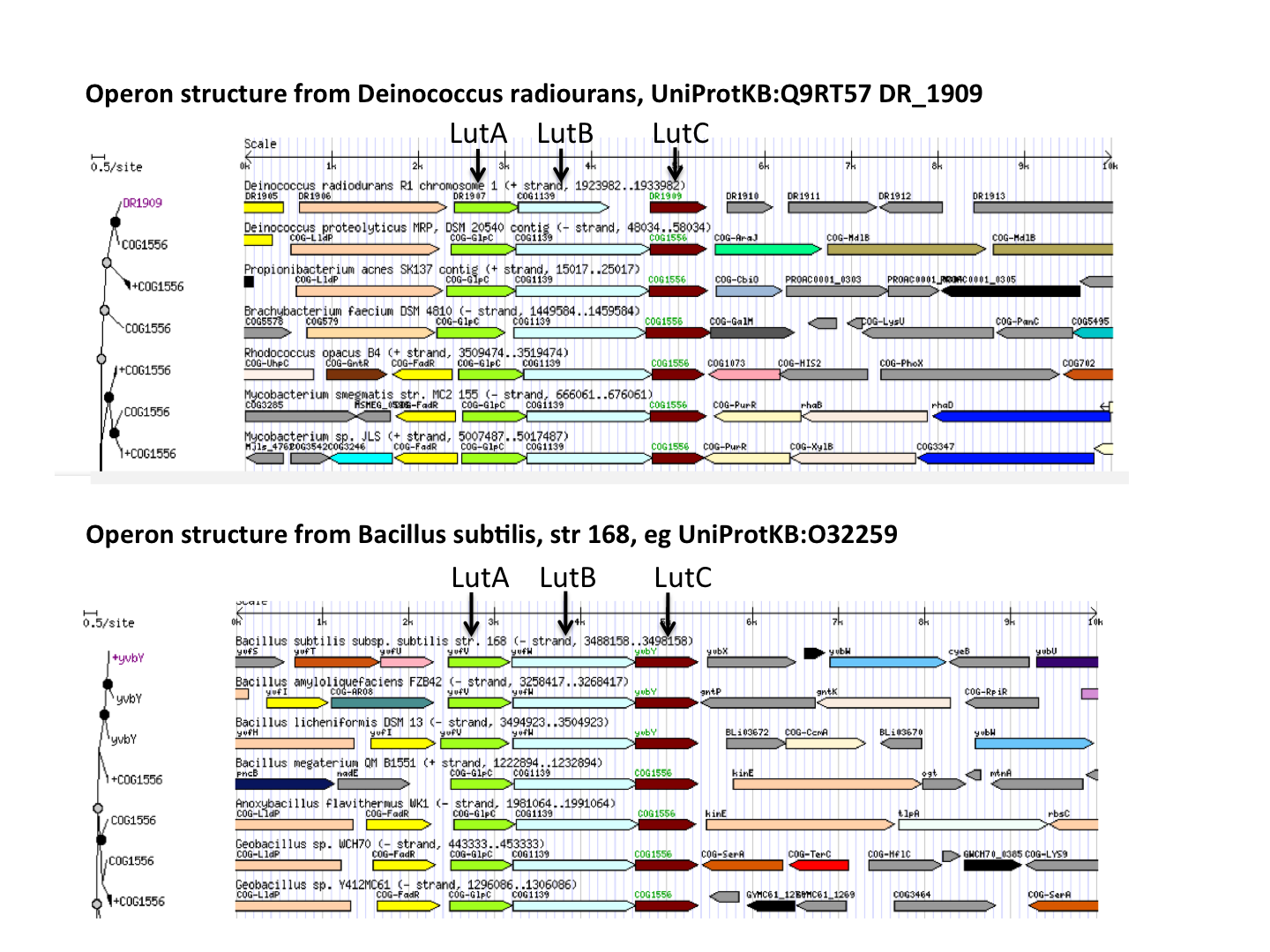
**

# Reference

1. Altschul SF, Gish W, Miller W, Myers EW, Lipman DJ (1990) Basic local alignment search tool. J Mol Biol 215: 403-410.

2. Katoh K, Kuma K, Toh H, Miyata T (2005) MAFFT version 5: improvement in accuracy of multiple sequence alignment. Nucleic Acids Res 33: 511-518.

3. Waterhouse AM, Procter JB, Martin DM, Clamp M, Barton GJ (2009) Jalview Version 2--a multiple sequence alignment editor and analysis workbench. Bioinformatics 25: 1189-1191.

4. Alm EJ, Huang KH, Price MN, Koche RP, Keller K, et al. (2005) The MicrobesOnline Web site for comparative genomics. Genome Res 15: 1015-1022.
