# Supplementary material for: LUD, a new protein domain associated with lactate utilization
Source: BMC Bioinformatics. 2013 Nov 26;14:341. doi: 10.1186/1471-2105-14-341 (PMC3924224; doi:10.1186/1471-2105-14-341)
Supplement: Additional file 2 — Experimental Details [PDB:2G40]. This section contains experimental details as well as structural and refinement statistics. [file 1471-2105-14-341-S2.docx]

Additional file 2

# Methods

## Data collection, structure solution, and refinement

X-ray diffraction data were collected from two different crystals of LutC. The first set of x-ray diffraction data were collected at the Stanford Synchrotron Radiation Lightsource (SSRL) on beamline 1-5 at a resolution of 2.10 Å. Data sets were collected at 100 K using an ADSC Quantum 4 CCD detector. X-ray diffraction data were collected from a single crystal at wavelengths corresponding to the high energy remote (λ_1_) and inflection (λ_2_), and peak (λ_3_) of a three-wavelength selenium multi-wavelength anomalous diffraction (MAD). The data were integrated and scaled using the XDS and XSCALE programs respectively[[1](#_ENREF_1),[2](#_ENREF_2)]. Data statistics are summarized in Table 1. The selenium substructures for the three proteins were solved with SHELXD[[3](#_ENREF_3)] and the MAD phases were refined with *autoSHARP* [[4](#_ENREF_4)]. Iterative automated model building was performed with Arp/Warp[[5](#_ENREF_5)] from density-modified electron density at a resolution of 2.10 Å from density-modified electron density. Model completion was performed using the interactive computer-graphics program COOT[[6](#_ENREF_6)]. The initial refinement of the coordinates using REFMAC ver 5.2.0005 at a resolution of 2.10 Å was restrained against the MAD phases. X-ray diffraction data were collected at an enhanced resolution of 1.7 Å from a second native crystal (Met instead of SeMet) of LutC isomorphous with the first crystal (same unit cell dimensions and space group) at the Advanced Light Source (ALS) on beamline 5.0.3. The refinement of the coordinates at an enhanced resolution of 1.7 Å was also performed with REFMAC ver 5.2.0005 with the experimental phases from the first crystal of the SeMet-substituted protein that was used for initial phase determination by MAD.

## Validation and deposition

The quality of the crystal structure was analyzed using the JCSG Quality Control server (see <http://smb.slac.stanford.edu/jcsg/QC/>). This server verifies: the stereochemical quality of the model using AutoDepInputTool[[7](#_ENREF_7)], MolProbity[[8](#_ENREF_8)], and WHATIF 5.0[[9](#_ENREF_9)]; agreement between the atomic model and the data using SFcheck 4.0[[10](#_ENREF_10)], and RESOLVE[[11](#_ENREF_11)]; the protein sequence using CLUSTALW[[12](#_ENREF_12)]; atom occupancies using MOLEMAN2.0[[13](#_ENREF_13)]; and consistency of NCS pairs. It also evaluates differences in R_cryst_/R_free_, expected R_free_/R_cryst_, and maximum/minimum B-values by parsing the refinement log-file and PDB header. Protein quaternary structure analysis used the EBI PISA server[[14](#_ENREF_14)]. Atomic coordinates and experimental structure factors have been deposited in the PDB and are accessible under the code 2G40.

**TABLE 1**
Summary of crystal parameters, data collection and refinement statistics for PDB 2G40

|  | | | | |  |
| --- | --- | --- | --- | --- | --- |
|  | λ_1_ Native | | λ_2_ MAD-Se | λ_3_ MAD-Se | λ_4_ MAD-Se |
|  | | | | |  |
| Data collection | |  |  |  |  |
| Space group | | P4_3_2_1_2 |  |  |  |
| Unit cell parameters (Å) | | a=53.41 Å b=53.41 c=118.31 Å | | |  |
| Wavelength (Å) | | 1.0000 | 0.91838 | 0.97931 | 0.97836 |
| Resolution range (Å) | | 27.28-1.70  (1.74-1.70) | 27.5-2.10  (2.15-2.10) | 27.52-2.10  (2.16-2.10) | 27.47-2.10  (2.15-2.10) |
| No. of observations | | 249,747 | 74,641 | 72,116 | 72,033 |
| No. of unique reflections | | 19,674 | 10,920 | 10,932 | 10,861 |
| Completeness (%) | | 99.8(98.0) | 99.9 (98.6) | 99.9 (99.1) | 99.6 (94.9) |
| Mean *I/σ (I)* | | 25.5(3.9) | 14.2 (4.1) | 14.1 (3.6) | 14.0 (3.1) |
| *R_merge_* on *I*^†^ (%) | | 6.9(53.8) | 9.3 (46.8) | 8.6 (44.8) | 9.6 (53.2) |
| *R_meas_* on *I*^‡^ (%) | | 7.2(58.2) | 10.0(50.6) | 9.3 (50.4) | 21.9 (138.4) |
| Model and refinement Statistics | | |  |  |  |
| Resolution range (Å) | | 27.28-1.70 |  |  |  |
| No. of reflections (total) | | 19,611^§^ |  |  |  |
| No. of reflections (test) | | 984 |  |  |  |
| Completeness (%) | | 99.8 |  |  |  |
| Cutoff criteria | | \|F\|>0 |  |  |  |
| *R_cryst_*^¶^ | | 0.178 |  |  |  |
| *R_free_*^¶^ | | 0.220 |  |  |  |
| Stereochemical parameters | |  |  |  |  |
| Restraints (RMSD observed) | | |  |  |  |
| Bond angles (º) | | 1.32 |  |  |  |
| Bond lengths (Å) | | 0.011 |  |  |  |
| Average isotropic *B* value^††^ (Å^2^) | | 19.50 |  |  |  |
| ESU^‡‡‡^ based on *R_free_* (Å) | | 0.103 |  |  |  |
| Protein residues/ atoms | | 164 / 1216 |  |  |  |
| Waters | | 132 |  |  |  |
|  | | | | |  |
| Values in parentheses are for the highest resolution shell. ^†^ *R_merge_* = Σ*_hkl_*Σ*_i_*\|*I_i_(hkl) - (I(hkl))*\|/Σ*_hkl_* Σ*_i_(hkl)*.  ^‡^ *R_meas_* = Σ*_hkl_*[*N/(N*-1)]^1/2^Σ*_i_*\|*I_i_(hkl) - (I(hkl))\|/*Σ*_hkl_*Σ*_i_I_i_(hkl)* [[15](#_ENREF_15)].  ^§^ Typically, the number of unique reflections used in refinement is slightly less than the total number that were integrated and scaled. Reflections are excluded owing to negative intensities and rounding errors in the resolution limits and unit-cell parameters.  ^¶^ *R_cryst_* = Σ*_hkl_*\|\|*F*_obs_\| - \|*F*_calc_\|\|/Σ*_hkl_*\|*F*_obs_\|, where *F*_calc_ and *F*_obs_ are the calculated and observed structure-factor amplitudes, respectively. *R_free_* is the same as *R_cryst_* but for 5.0% of the total reflections chosen at random and omitted from refinement.  ^††^ This value represents the total *B* that includes TLS and residual *B* components.  ^‡‡‡^ Estimated overall coordinate error[[16](#_ENREF_16)]. | | | | |  |

# References

1. Kabsch W (1993) Automatic processing of rotation diffraction data from crystals of initially unknown symmetry and cell constants. Journal of Applied Crystallography 26: 795-800.

2. Kabsch W (2010) Xds. Acta Crystallogr D Biol Crystallogr 66: 125-132.

3. Schneider TR, Sheldrick GM (2002) Substructure solution with SHELXD. Acta Crystallogr D Biol Crystallogr 58: 1772-1779.

4. Vonrhein C, Blanc E, Roversi P, Bricogne G (2007) Automated structure solution with autoSHARP. Methods Mol Biol 364: 215-230.

5. Langer G, Cohen SX, Lamzin VS, Perrakis A (2008) Automated macromolecular model building for X-ray crystallography using ARP/wARP version 7. Nat Protoc 3: 1171-1179.

6. Emsley P, Cowtan K (2004) Coot: model-building tools for molecular graphics. Acta Crystallogr D Biol Crystallogr 60: 2126-2132.

7. Yang H, Guranovic V, Dutta S, Feng Z, Berman HM, et al. (2004) Automated and accurate deposition of structures solved by X-ray diffraction to the Protein Data Bank. Acta Crystallogr D Biol Crystallogr 60: 1833-1839.

8. Chen VB, Arendall WB, 3rd, Headd JJ, Keedy DA, Immormino RM, et al. (2010) MolProbity: all-atom structure validation for macromolecular crystallography. Acta Crystallogr D Biol Crystallogr 66: 12-21.

9. Vriend G (1990) WHAT IF: a molecular modeling and drug design program. J Mol Graph 8: 52-56, 29.

10. Vaguine AA, Richelle J, Wodak SJ (1999) SFCHECK: a unified set of procedures for evaluating the quality of macromolecular structure-factor data and their agreement with the atomic model. Acta Crystallogr D Biol Crystallogr 55: 191-205.

11. Terwilliger T (2004) SOLVE and RESOLVE: automated structure solution, density modification and model building. J Synchrotron Radiat 11: 49-52.

12. Chenna R, Sugawara H, Koike T, Lopez R, Gibson TJ, et al. (2003) Multiple sequence alignment with the Clustal series of programs. Nucleic Acids Res 31: 3497-3500.

13. Kleywegt GJ (1997) Validation of protein models from Calpha coordinates alone. J Mol Biol 273: 371-376.

14. Krissinel E, Henrick K (2007) Inference of macromolecular assemblies from crystalline state. J Mol Biol 372: 774-797.

15. Diederichs K, Karplus PA (1997) Improved R-factors for diffraction data analysis in macromolecular crystallography. Nat Struct Biol 4: 269-275.

16. Cruickshank DW (1999) Remarks about protein structure precision. Acta Crystallogr D Biol Crystallogr 55: 583-601.
